# Supplementary material for: Overexpression of DGAT2 Regulates the Differentiation of Bovine Preadipocytes
Source: Animals (Basel). 2023 Mar 29;13(7):1195. doi: 10.3390/ani13071195 (PMC10093762; doi:10.3390/ani13071195)
Supplement: Supplementary file 1 [file animals-13-01195-s001.zip › animals-2248275-supplementary.pdf]

Overexpression of DGAT2 Regulates the Differentiation of Bovine Preadipocytes

Pan-Pan Guo , Xin Jin, Jun-Fang Zhang, Qiang Li, Chang-Guo Yan and Xiang-Zi Li 1,*

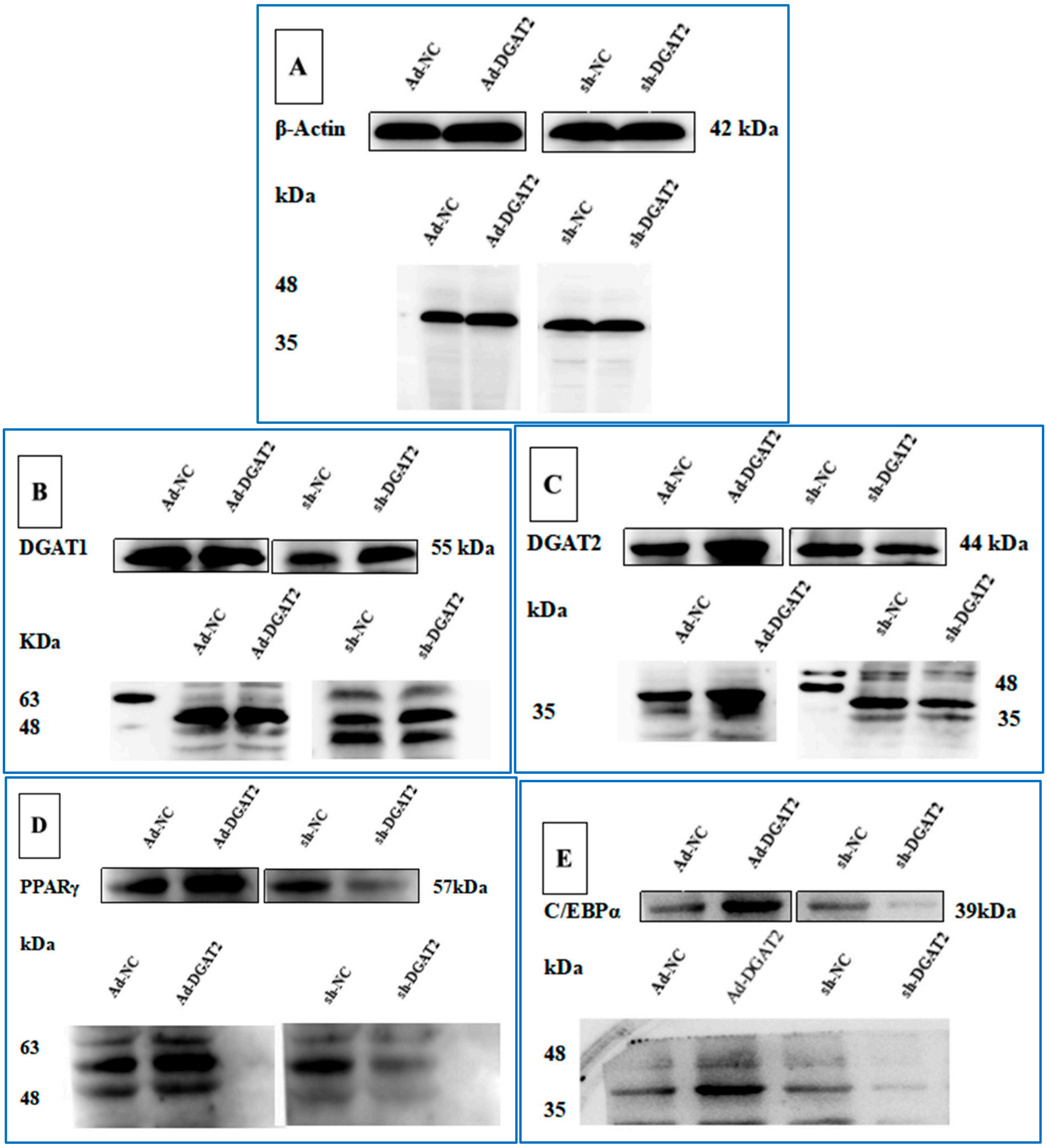

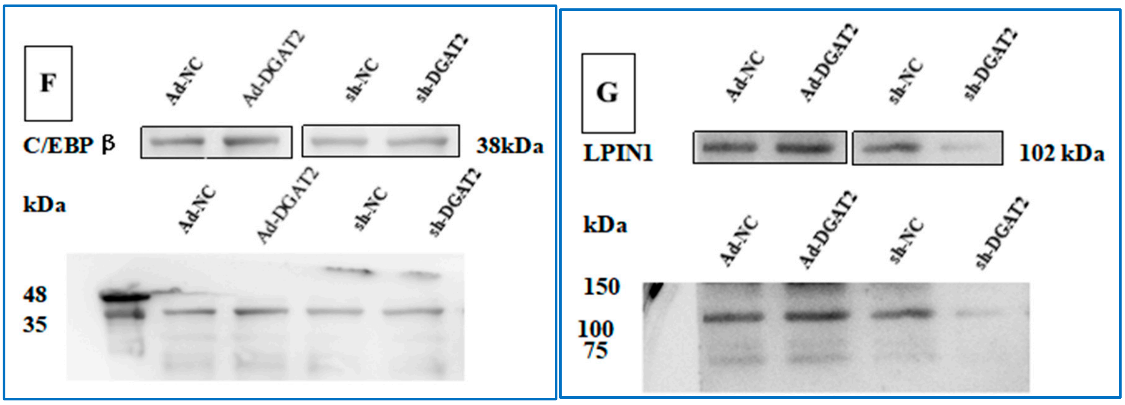

Figure S1. The original figure of the upper blot membrane of Figure 4D and 5D.The original western blot of β-actin (A),DGAT1 (B), DGAT2 (C), PPARγ (D),C/EBPα (E), C/EBPβ (F), LPIN1(G) protein in adipocytes transfected with Ad-DGAT2/Ad-NC and sh-DGAT2/sh-NC.

Table S1: Sequence information of PCR primers and Full length of CDS region sequence of DGAT2 gene in Yanbian cattle

Genes	Primer sequences(5→3′)	Product size/bp	Annealing temperature/°C	GenBank Accession No.
DGAT2	F:TGAACCGGGACACCATAGACTACTTG<br>R:TCAGTTCACCTCCAGGACCTCG	1086	65	NM_205793.2
Sequence	ATGAAGACCCTCATAGCCGCCTACTCCGGGGTCCTGCGAGGCACTGGCTCCAGCATCCT<br>CTCTGCCCTCCAGGACCTGTTTTCTGTCACTTGGCTCAATAGGTCCAAGGTAGAGAAGCA<br>GCTCCAAGTCATCTCGGTGCTACAATGGGTCCTGTCTTTCCTCGTGCTGGGAGTGGCCTG<br>CAGCGTCATCCTCATGTACACATTCTGCACCGATTGCTGGCTCATTGCCGTGCTCTACTTC<br>ACCTGGCTGGTGTTTGACTGGAACACACCCAAGAAAGGTGGCAGGAGGTCACAGTGGG<br>TCCGAAACTGGGCTGTGTGGCGCTACTTTCGAGACTACTTTCCCATTCAGCTGGTGAAGA<br>CACACAACTTACTGACCAGCAGGAACTACATCTTTGGGTACCATCCCCATGGCATCATG<br>GGCCTGGGTGCCTTCTGCAACTTCAGCACAGAGGCCACAGAAGTAAGCAAGAAGTTCCC<br>TGGCATAAGGCCCTACCTGGCCACGCTGGCCGGCAACTTCCGGATGCCAGTGCTGCGGG<br>AGTACCTGATGTCTGGAGGCATCTGCCCAGTGAACCGGGACACCATAGACTACTTGCTTT<br>CAAAGAATGGGAGTGGCAATGCCATCATCATCGTGGTGGGGGGCGCGGCTGAATCCCTG<br>AGCTCCATGCCCGGCAAGAATGCAGTCACCCTGCGCAATCGCAAGGGCTTTGTGAAACT<br>GGCCCTGCGCCATGGAGCCGACCTGGTTCCCACCTACTCCTTTGGGGAGAATGAGGTGT<br>ACAAGCAGGTGATCTTTGAGGAGGGCTCCTGGGGCCGGTGGGTGCAGAAGAAGTTCCA<br>GAAGTACATTGGCTTTGCCCCATGCATCTTCCATGGTCGAGGCCTCTTCTCCTCTGACACC<br>TGGGGGCTGGTGCCCTACTCCAAGCCCATCACCACTGTCGTGGGCGAGCCCATTACCAT<br>CCCCAGGCTGGAGCGCCCGACGCAGCAGGACATCGACCTGTACCACGCCATGTACGTG<br>CAAGCCCTGGTGAAGCTCTTCGACCAGCATAAGACCAAGTTCGGCCTCCCGGAGACCGA<br>GGTCCTGGAGGTGAACTGA			

Table S2. Information of clean data.

Sample	Trimmed_Read_Number	Trimmed_Bases	Useful_read%	Useful_bases%
Ad-DGAT2-1	41957774	6293666100	90.49	90.49
Ad-DGAT2-2	46692432	7003864800	90.25	90.25
Ad-DGAT2-3	45059410	6758911500	89.56	89.56
Ad-NC-1	46123688	6918553200	90.33	90.33
Ad-NC-2	48540484	7281072600	90.97	90.97
Ad-NC-3	47456602	7118490300	91.47	91.47
sh-NC-1	47383396	7107509400	90.52	90.52
sh-NC-2	43421586	6513237900	90.92	90.92
sh-NC-3	41991732	6298759800	90.15	90.15
sh-DGAT2-1	50881030	7632154500	89.97	89.97
sh-DGAT2-2	41109628	6166444200	90.9	90.9
sh-DGAT2-3	43272494	6490874100	88.08	88.08

Table S3. Summary of sequencing dada and reference genome comparison.

Sample	Clean Reads	Total Mapped	Multiple Mapped	Uniquely Mapped	Map Events	Mapped to Gene	Mapped to InterGene	Mapped to Exon
sh-NC-1	47383396	45346415 (95.70%)	1243402 (2.74%)	44103013 (97.26%)	44103013	37110627 (84.15%)	6992386 (15.85%)	33656706 (90.69%)
sh-NC-2	43421586	41492311 (95.56%)	1132667 (2.73%)	40359644 (97.27%)	40359644	33788214 (83.72%)	6571430 (16.28%)	30673434 (90.78%)
sh-NC-3	41991732	40078263 (95.44%)	1032503 (2.58%)	39045760 (97.42%)	39045760	32844563 (84.12%)	6201197 (15.88%)	29889150 (91.00%)
Ad-NC-1	46123688	43994995 (95.38%)	1400082 (3.18%)	42594913 (96.82%)	42594913	35641438 (83.68%)	6953475 (16.32%)	32542118 (91.30%)
Ad-NC-2	48540484	46377795 (95.54%)	1383335 (2.98%)	44994460 (97.02%)	44994460	37616399 (83.60%)	7378061 (16.40%)	34420035 (91.50%)
Ad-NC-3	47456602	45389952 (95.65%)	1643704 (3.62%)	43746248 (96.38%)	43746248	36577634 (83.61%)	7168614 (16.39%)	33848556 (92.54%)
Ad-DGAT2-1	41957774	40124294 (95.63%)	1200506 (2.99%)	38923788 (97.01%)	38923788	32390894 (83.22%)	6532894 (16.78%)	29422088 (90.83%)
Ad-DGAT2-2	46692432	44489772 (95.28%)	1303662 (2.93%)	43186110 (97.07%)	43186110	35978292 (83.31%)	7207818 (16.69%)	32729531 (90.97%)
Ad-DGAT2-3	45059410	43080975 (95.61%)	1329255 (3.09%)	41751720 (96.91%)	41751720	34822208 (83.40%)	6929512 (16.60%)	31740502 (91.15%)
sh-DGAT2-1	50881030	48642046 (95.60%)	1740615 (3.58%)	46901431 (96.42%)	46901431	38936622 (83.02%)	7964809 (16.98%)	35376210 (90.86%)
sh-DGAT2-2	41109628	39315425 (95.64%)	1004356 (2.55%)	38311069 (97.45%)	38311069	31978328 (83.47%)	6332741 (16.53%)	29086478 (90.96%)
sh-DGAT2-3	43272494	41349412 (95.56%)	1126742 (2.72%)	40222670 (97.28%)	40222670	33893908 (84.27%)	6328762 (15.73%)	30933390 (91.27%)
